# Transient ischemic attack in the twenty first century: is it still a useful construct?

**DOI:** 10.3389/fstro.2023.1241649

**Published:** 2024-01-19

**Authors:** Louis R. Caplan

**Affiliations:** Neurology, Beth Israel deaconess Medical Center, Boston, MA, United States

**Keywords:** transient ischemic attack, minor stroke, care, definition, cerebrovascular disease

## Abstract

Some argue that the term *transient ischemic attack* (TIA) has become obsolete in the current age of advanced modern technology. Let us look back and analyze the term, its support and detraction, and its potential continued usefulness. The best way to begin is a review of the history of the term.

## Origin and early history of the term

At the midpoint of the twentieth century, there were no organizations devoted solely to stroke, no stroke-oriented journals, and no physicians who professed to specialize in stroke. Stroke was a non-entity. In 1954, the American Heart Association sponsored a cerebrovascular disease meeting held in Princeton, New Jersey. Those who attended this first conference were mostly internists and cardiologists. A second conference was held in January 1957 (Millikan, 1957). The National Institutes of Health (NIH), a major focal point of medical research in the United States, acknowledged the lack of research and knowledge about stroke and its treatment. Two component organizations of the NIH, the National Institute of Neurological Diseases and Blindness and the National Heart Institute, appointed an *ad hoc* committee titled the Joint Council Subcommittee on Cerebrovascular Diseases. This committee functioned from 1961 to 1972 as the NIH focal point for planning and funding subsequent Princeton Conferences on Cerebrovascular Diseases.

At the first two Princeton Conferences, in 1954 and later in 1956, warning spells before strokes were discussed. Sir William Gowers and Sir William Osler had made comments about transient episodes in their neurology and medical texts circa 1900. During the first Princeton Conference, Miller Fisher shared his experience with temporary warning events in patients who later developed strokes:

*If a satisfactory history can be obtained... one finds in a great many cases that there had been a warning prior to the stroke. The warnings may go back weeks or months. There may have been only one or as many as 500. Some of these very interesting cases lying in the wards are described simply as “had a stroke this morning” but in going into the details many premonitory symptoms may be elicited. I have seen a man with eight attacks a day for 2 months, each attack characterized by numbness around the lip, numbness of the thumb and index finger, and drooping of the lip. Attacks occurred in physicians' offices. Finally, the patient awakened one morning with a massive hemiplegia from which there has been practically no recovery* (Cerebral Vascular Diseases, 1954).

During the first two Princeton Conferences, various terms for these temporary episodes were suggested: *intermittent vascular insufficiency, ischemic recurrent attacks, recurrent focal cerebral ischemic attacks, transient cerebral ischemia*, and *transient ischemic attacks*. At the Fourth Princeton Conference held in 1965, the conference attendees agreed on the term *transient ischemic attack* (TIA) (Siekert and Whisnant, 1965). An *ad hoc* committee on cerebrovascular disease in 1975 used the following definition: “Transient ischemic attack is defined as a “cerebral dysfunction of ischemic nature lasting no longer than 24 h with a tendency to recur.” (Millikan, 1975) The 24-h definition was arbitrarily chosen.

## Problems with the early definition and later progress

During the ensuing decades, two main issues surfaced. The first was that, although most episodes were much shorter than 24 h, some episodes lasted longer than a day. More importantly, when computed tomography and later magnetic resonance imaging scans were performed, brain infarcts were found in areas that correlated with the transient symptoms during the attacks. Terms and acronyms were proffered for longer lasting deficits: *stroke with full recovery*, SFR; *reversible ischemic neurological deficit*, RIND; *partially reversible neurological deficit*, PRIND; *stroke in evolution*; and *completed stroke*. Later, the term *cerebral infarction with transient signs* was proposed (Waxman and Toole, 1983).

The second problem was that the term *TIA* was not often recognized or understood by the public, patients, or even physicians. More importantly, early and even late studies showed that neurologists used the term variably (Kraaijeveld et al., 1984). Studies of interobserver use of the term showed that many non-neurologists (Ferro et al., 1996) and even fellowship-trained neurologists (Castle et al., 2010) had low interobserver agreement regarding the diagnosis of TIA. Some patients had other neurological conditions that were not related to brain ischemia. Many had stroke mimics.

A group of nationwide U.S. stroke experts met regularly from 2000 to 2002 to discuss redefining TIA terminology and to reach a consensus on a new definition, treatment guidelines, and risk factors (Albers et al., 2002; Easton et al., 2004). They chose a new definition of TIA consonant with available information from a series of cases and from brain imaging: “Transient ischemic attack is a brief period of neurological dysfunction caused by focal brain or retinal ischemia with clinical symptoms typically lasting less than an hour, and without evidence of acute infarction” (Albers et al., 2002). After the publication of a 2009 position article from the American Heart/American Stroke Organizations, TIA was defined as a neurological deficit typically lasting < 1 h with no evidence of stroke on imaging (Easton et al., 2009).

Clinicians sought information about how often patients with TIAs had strokes. How soon after the last TIA? Data showed that strokes were very common soon after a TIA. One study showed that the timing was highly consistent across studies, with 17% of TIAs occurring on the day of the stroke, 9% on the previous day, and 43% at some point during the 7 days prior to the stroke (Rothwell and Warlow, 2005). This information meant that not only was TIA an important warning of stroke but also that there was urgency since the timing of evaluation and treatment was important.

Was the frequency of stroke after TIA different if there was imaging evidence of a relevant brain infarct? To answer these and other questions, a large TIA registry was organized in Europe. The TIA registry recruited patients who had had a TIA or minor stroke within the previous 7 days and were evaluated at sites that had systems dedicated to the urgent evaluation of patients. Between 2009 through 2011, the registry enrolled 4,789 patients at 61 sites in 21 countries. More than 75% of patients were evaluated by stroke specialists within 24 h after symptom onset (Amarenco et al., 2016, 2018). Patients with imaging evidence of relevant brain infarction (technically minor strokes) had a higher frequency of strokes than those with negative studies (Giles et al., 2011).

Because the etiology and evaluation of patients with TIAs and minor strokes were identical and each had an important risk of developing new brain infarction, Kidwell and Warach (2003) suggested using the term *acute ischemic cerebrovascular syndrome* to include both TIA and minor stroke patients. This term did not gain much popularity and was seldom used.

## Assessment

There seems to be a general consensus regarding several conclusions:

- Patients with TIAs have a much higher risk of stroke than those who do not have TIAs.
- The risk of stroke is highest during the first hours and days after a TIA.
- TIAs and ischemic strokes have the same etiologies.
- The prognosis for developing a stroke and treatment after a TIA depends on the cause (cardiac-cranio-cerebral arterial hematological disease).

If the term *TIA* was subjected to a grade, it would likely be approximately a C+. However, the term *TIA* does have a long precedent, nearly half a century. Nevertheless, the term is still in wide use among physicians. However, the recognition that a patient had a TIA stimulates an urgent evaluation and often an urgent consultation with a neurologist or other experienced stroke clinician. The fact that some TIAs are actually brain infarcts and some prove not to be attributable to ischemia does not really detract from the necessity and urgency of evaluation. Therefore, although the term *TIA* is flawed, it is still very useful to retain. There is no real, practical rival term to replace it, which would improve patient care.

## Author contributions

The author confirms being the sole contributor of this work and has approved it for publication.
